# *Giardia duodenalis* in Alpine (*Rupicapra rupicapra rupicapra*) and Apennine (*Rupicapra pyrenaica ornata*) chamois

**DOI:** 10.1186/s13071-015-1243-1

**Published:** 2015-12-21

**Authors:** Claudio De Liberato, Federica Berrilli, Marianna Marangi, Maristella Santoro, Tiziana Trogu, Lorenza Putignani, Paolo Lanfranchi, Francesco Ferretti, Stefano D’Amelio, Annunziata Giangaspero

**Affiliations:** Istituto Zooprofilattico Sperimentale del Lazio e della Toscana “M. Aleandri”, 00178 Roma, Italy; Dipartimento di Medicina sperimentale e Chirurgia, Università degli Studi di Roma ‘Tor Vergata’, 00133 Roma, Italy; Dipartimento di Scienze Agrarie, degli Alimenti e dell’Ambiente, Università degli Studi di Foggia, 71121 Foggia, Italy; Dipartimento di Scienze veterinarie e di Sanità pubblica, Università degli Studi di Milano, 20133 Milano, Italy; Unità di Parassitologia e Unità di Ricerca di Metagenomica, Bambino Gesù, Ospedale Pediatrico e Istituto di Ricerca, 00165 Roma, Italy; Dipartimento di Scienze della Vita, Università di Siena, 53100 Siena, Italy; Dipartimento di Sanità pubblica e Malattie infettive, Università degli Studi di Roma ‘Sapienza’, 00185 Roma, Italy

**Keywords:** *Giardia duodenalis*, *Rupicapra rupicapra rupicapra*, *Rupicapra pyrenaica ornata*, IF, *q*PCR, *end-point-*PCR

## Abstract

**Background:**

Although chamois *Rupicapra* spp. are the most abundant mountain ungulates in Europe, no data are available on the presence of *Giardia duodenalis* infecting these species.

**Methods:**

A total of 157 fecal samples from Alpine *Rupicapra rupicaprarupicapra* and Apennine *Rupicapra pyrenaicaornata* chamois were tested for the presence of *G. duodenalis* by immunofluorescence test, quantitative Real Time PCR and end-point PCR for genotype characterization.

**Results:**

*G. duodenalis* was detected in *R. r. rupicapra* and *R. p. ornata*, with a percentage value of 4.45 (5.82 and 1.85 %, respectively), and a cyst burden of up to 31,800 cysts/g of feces. Assemblages A/AI and E were identified in *R. r. rupicapra* and assemblage A/AIII in *R. p. ornata*.

**Conclusions:**

The present study represents the first record of *Giardia duodenalis* in *Rupicapra* spp., suggesting that these wild bovids can play an epidemiological role in environmental contamination and transmission of both zoonotic and non-zoonotic genotypes.

## Background

The flagellate *Giardia duodenalis* is one of the most common intestinal parasites in humans and several animal species worldwide [1, 2]. At present, eight assemblages have been genetically recognized (A-H), which differ in host specificity: zoonotic assemblages A and B infect humans and a wide variety of domestic and wild mammals; assemblages C and D are typically isolated from dogs; assemblage E is associated with hoofed livestock; assemblage F infects cats; assemblage G infects rats [3], and assemblage H infects marine mammals (pinnipeds) [4]. It is now believed that at least some of these assemblages should be considered “true species” [5, 6].

Given this great genetic heterogeneity, it is hard to determine the role of animals as a source for human infection, and *viceversa* [7]; this is possible only by performing detailed genetic analysis [8, 9], even as far as the sub-assemblage level. While the role of domestic animals (pets and livestock) in *G. duodenalis* epidemiology has been thoroughly studied, wild animals have only recently been considered as having a potential role. In addition to being a possible source of infection for humans, wild animals can be endangered by the spill-over of parasites from domestic animals and even people [10], especially in the case of small populations which are important for wildlife conservation [11].

*G. duodenalis* has been recorded in wild ungulates wordwide [8, 9, 12–15]. Most of these records refer to cervids, in which the presence of zoonotic and non-zoonotic genotypes have been documented (reviewed by [6]).

The chamois (Artiodactyla: Bovidae) is the most abundant mountain ungulate in Europe and the Near East. Two species are recognised in the genus *Rupicapra*: the Northern chamois, *Rupicapra rupicapra*, with seven subspecies, including the Alpine chamois *R. r. rupicapra*, and the Southern chamois, *Rupicapra pyrenaica*, with three subspecies, including the Apennine chamois *R. p. ornata* [16, 17].

Despite the geographical abundance of *Rupicapra* species in Europe, no data are available on the presence of *G. duodenalis* infecting chamois. This study aimed to determine the presence of *G. duodenalis,* and to quantify and characterize isolates from two subspecies of chamois: *R. r. rupicapra* living in northern Italy (Alps) and *R. p. ornata,* living in central Italy (Apennines).

## Methods

### Study areas, animals and collection of fecal samples

The study took place in three areas of Italy. The first is in the Lecchesi Alps and Pre-Alps, a hunting territory in Lombardy region, with an area of 253 km^2^ (45°59’N, 9°32’E), ranging from 300 to > 2000 m a.s.l. Here the *R. r. rupicapra* population in 2014 was estimated as 2077 individuals, giving an average population density of 8.2 chamois/km^2^ (Province of Lecco, unpublished data). The second area is in the Lepontine Alps, in the hunting district of Piedmont region (VCO2-Ossola Nord), with an extent of 72,740 ha (46°07‘ N, 8°17‘ E), ranging from 700 to 2400 m a.s.l. Here the chamois population was estimated as 1328 individuals in 2014, with an average density of 6.7 subjects/km^2^ [18]. The third area is in central Italy, in the Abruzzo, Lazio and Molise National Park (ALMNP, 497 km^2^, 41°44’N, 13°54’E), where samples were collected in Val di Rose, Mt. Meta, and Mt. Amaro sub-areas, ranging from 1650 to 2242 m a.s.l. In ALMNP, about 600 individuals of *R. p.ornata* were counted in 2014 [19], with local population densities of up to over 20 individuals/km^2^ [20, 21].

Between August 2013 and January 2014, 103 fresh fecal samples were collected from *R. r. rupicapra* chamois harvested during the hunting season, whereas the 54 fecal samples from *R. p. ornata* were collected from the ground soon after defecation. To avoid the risk of collecting feces from the same individuals, sampling was carried out on different slope sites and took into account, as far as possible, the animals’ sex and age. Fresh fecal specimens were collected and put into plastic bags, which were labeled and immediately packed in an insulated container with ice or cold packs. Specimens were then transported to the laboratory and processed within 1–3 days after collection.

### Giardia *detection*

All 157 faecal samples were examined using an immunofluorescence (IF) test for the detection of *G. duodenalis* (Kit Merifluor**®** Meridian Diagnostic, Cincinnati, OH, USA).

The positive samples were frozen and subjected to Real-Time PCR for quantitative analysis (*q*PCR) using the *SSU-rDNA* gene, and to end-point PCR for genotyping using two genes i.e. *SSU-rDNA* and *gdh*.

### DNA extraction

I.F. positive fecal samples were washed three times with PBS and subjected to 5 cycles of freezing with dry ice and thawing at 95 °C (5 min each step). DNA extraction was automatically performed by EZ1 BioRobot (Qiagen, Germany) following the manufacturer’s instructions. To obtain a high quality DNA, samples were purified by Amicon Ultra-0.5 Centrifugal Filter Unit (Millipore) following the manufacturer’s instructions.

### *Quantitative (q*PCR*) and melting curve analysis*

A sequence of *G. duodenalis SSU-rDNA* gene (KJ888984) [22] was selected as reference target to design the plasmid control. The pEX-A vector (Eurofins, MWG/Operon, Ebersberg, Germany) was used to insert a fragment of approximately 293 bp of *G. duodenalis SSU-rDNA* gene.

The concentration of the pEX-A2 *G. duodenalis* plasmid was measured using a fluorometer, and the corresponding copy number was calculated using the following equation:

pEX-A2 *G. duodenalis* (copy numbers) = 6.02 × 10^23^ (copy/mol) × pEX-A2 *G. duodenalis* amount (0.31 x 10^-5^ g/ml)/pEX-A2 *G. duodenalis* length (293 bp + 2450 bp) × 660 (g/mol/bp) [23].

Ten-fold serial dilutions of the pEX-A2 *G. duodenalis* plasmid (from 1.03 x 10^7^ to 1.03 x 10^-3^ copies/μl) were used to assess the sensitivity, repeatability and reproducibility parameters of the assay, and to determine the quantity of the unknown samples based on linear regression calculations of the standard curve.

Amplifications and melting analysis were performed in the CFX-96 Real Time Instrument (BioRad, Italy). *G. duodenalis* ssRNA primers were GiarF (5’- GAC GCT CTC CCC AAG GAC-3’) and GiarR (5’- CTG CGT CAC GCT GCT CG-3’) [24].

The PCR mixture (final volume 20 μl) contained 1 μl of the plasmid-based control (or 5 μl of genomic DNA sample from 1 to 5 ng), 5X EvaGreen® Reagent (cat. No. 172–5201; BioRad, Italy) and 0.5 μM final concentration of each forward and reverse primer. Samples without genomic DNA (negative controls) were included in each PCR run. The cycling conditions in a CFX-96 thermocycler (BioRad) were as follows: initial denaturation at 98 °C for 2 min, followed by amplification for 35 cycles of 98 °C for 5 s and 55.6 ° C for 15 s.

Fluorescence data were collected at the end of each cycle as a single acquisition. After amplification, the PCR products were melted by raising the temperature from 70 to 95 °C, with an increment of 0.5 °C/5 s, in order to denature and re-anneal before the high resolution melting; changes in fluorescence were recorded with changes in temperature (d*F*/d*T*) and plotted against changes in temperature. The resolution melting curve (MC) profile was then analyzed using Precision Melt Analysis™ software version 1.2, with fluorescence (MC) normalization by selecting the linear region before and after the melting transition. The melting temperature (*Tm*) was interpolated from the normalized data as the temperature at 50 % fluorescence. Samples’ melting curves were distinguished by plotting the fluorescence difference between normalized melting curves. *Tm* and standard deviation (*SD*) were recorded for each positive control.

Test-positive samples were identified on the basis of a single melting peak, which was consistent with that of the homologous plasmid control. The melting peak was 92.50 °C for *G. duodenalis SSU-rDNA*.

The copy number for each positive sample was calculated by relating the *Ct* mean value of each sample obtained in *q*PCR to a standard curve obtained from the respective plasmid control. Since the number of copies of the *SSU-rDNA* gene ranges from 60 to 130 in one *Giardia* nucleus [25], we considered an average of 95 copies in one nucleus and a total for 4 nuclei of 380 copies in one cyst. The number of cysts in each sample was calculated as the number of copies obtained in *q*PCR divided by 380 in 1 μl and then in 100 μl (since the volume of DNA after extraction is 100 μl). Finally, since the number of cysts in each sample was obtained in 200 mg of fecal sample, the results were transformed for cysts per gram (CPG) with the formula: number of cysts obtained in 200 mg of fecal sample X 5.

### End-point PCR

A nested PCR was performed to amplify a 130 bp region from the *SSU-rDNA* gene, using the primers RH4 and RH11 for the first step, and the primers GiarR and GiarF in the second amplification round, as used for *q*PCR [24]. An additional analysis was carried out by using a semi-nested PCR to amplify a 432 bp fragment with the primers GDHeF and GDHiR in the primary reaction, and GDHiF and GDHiR in the secondary [26]. In all PCR reactions, positive (*Giardia* DNA) and negative (no template added) controls were added. All PCRs were carried out in a 25 μL volume containing 12.5 μL PCR master mix 2X (Promega), 5 μL template DNA, 0.6 mM of each primer and 0.1 mM BSA, 4 % dimethyl sulfoxide (DMSO), and were performed in a TProfessional Basic Thermocycler (Biometra GmbH, Göttingen, Germany). PCR products were visualized by electrophoresis on 1 % agarose gel stained by SYBR Safe DNA gel stain (Invitrogen). Amplicons were purified using the mi-PCR Purification Kit (Metabion GmbH). Both strands were sequenced by Bio-Fab Research s.r.l. (Rome, Italy). Sequences were edited with FinchTV 1.4 software (Geospiza, Inc, Seattle, WA, USA). To assign *Giardia* isolates to the correct assemblage, a comparison of the *SSU-rDNA* sequences by multiple alignments was performed using ClustalW2 software against known sequences available in GenBank. To test the significance of the results and to identify the sub-assemblage, a phylogenetic analysis was performed using MEGA6 software to compare the *SSU-rDNA* and *gdh* sequences with those of reference strains from different hosts. The best-fit model and parameters for tree construction were selected using the jModeltest software by the Akaike Information Criterion (AIC).

## Results

*G. duodenalis* was detected by microscopy in seven out of the 157 fecal samples examined, (4.45 %; CI = 1.8–9.1). In *R. r. rupicapra* and *R. p. ornata*, a percentage of 5.82 % (6/103) (CI = 2.2–12.5) and 1.85 % (1/54) (CI = 0.5–9.8) were registered, respectively. All samples which tested positive to microscopy were also positive to *q*PCR and end-point PCR to one or both genes (i.e. *SSU-rDNA*, *gdh*). Overall, two assemblages i.e. A (with sub-assemblages AI and AIII) and E were identified (Table 1), and combined analysis of the two loci revealed no discrepancies in assemblage assignment.

**Table 1:** Number of individual chamois (*Rupicapra r. rupicapra* and *Rupicapra p. ornata*) investigated and test-positive to *Giardia duodenalis* by microscopy, *q*PCR and end-point PCR analysis

Collection sites	Species	Animal number	*Giardia duodenalis*			Assemblage/Sub-assemblage	No. of CPG
			Microscopy	*q*PCR	PCR		
ALPS	*Rupicapra r. rupicapra*	1–3	_	_	_	_	_
		4	+	+	+	A	382
		5–25	_	_	_	_	_
		26	+	+	+	E	326
		27–31	_	_	_	_	_
		32	+	+	+	E	587
		33–54	_	_	_	_	_
		55	+	+	+	A	263
		56–70	_	_	_	_	_
		71	+	+	+	A/AI	31,800
		72–92	_	_	_	_	_
		93	+	+	+	E	11,200
		94–103	_	_	_	_	_
APENNINES	*Rupicapra p. ornata*	1–9	_	_	_	_	_
		10	+	+	+	A/AIII	618
		10–54	_	_	_	_	_

*CPG* cysts per gram of faeces calculated by *q*PCR

**Fig. 1:**
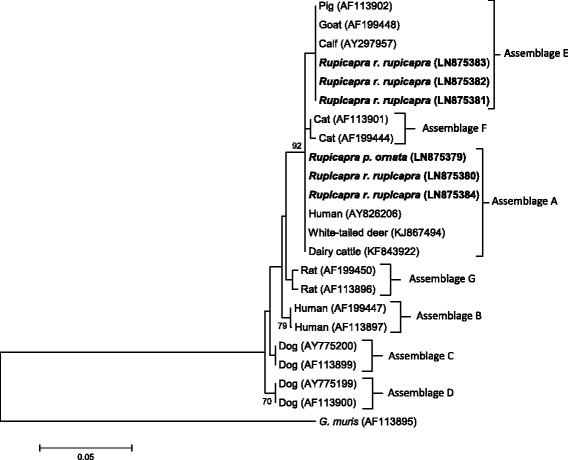
Neighbor-Joining tree of the *SSU-rDNA Giardia* sequences. Six sequences from the present study (in bold) and 16 reference sequences representing assemblages A-G were included in the analysis for comparative purposes. Accession numbers of publicly available reference sequences are indicated. The evolutionary distances were computed using the Tamura 3-parameter method and a bootstrap test based on 1000 replicates. Bootstrap values at nodes <70 are not indicated. *Giardia muris* (AF113895) represents the outgroup

At the *SSU-rDNA* locus, *G. duodenalis* assemblage A sequences (chamois no.s 10, 55, and 71) were identical to those from different hosts, including white-tailed deer in the USA (Genbank accession number KJ867494) previously reported by [27], dairy cattle (KF843922) in China [28] and Dutch patients (AY826206) [29] (Fig. 1). The isolates from chamois no.s 26, 32 and 93 matched with several assemblage E isolates from livestock (100 % similarity). These assignments were confirmed by the phylogenetic analysis as evidenced in Fig. 1.

**Fig. 2:**
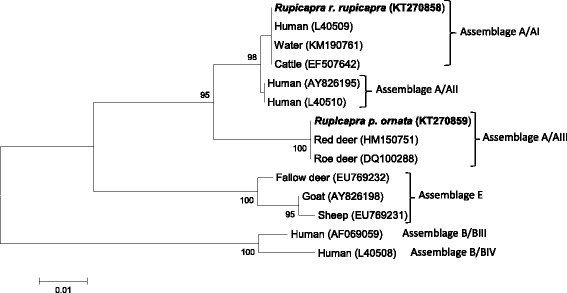
Neighbor-Joining tree of the *gdh Giardia* sequences. Two sequences from the present study (in bold) and 12 reference sequences representing assemblage/sub-assemblages A/AI, A/AII, A/AIII, B/BIII, B/BIV and assemblage E were included in the analysis for comparative purposes. Accession numbers of publicly available reference sequences are indicated. The evolutionary distances were computed using the Tamura 3-parameter method and a bootstrap test based on 1000 replicates. Bootstrap values at nodes <70 are not indicated

Phylogenetic analysis of *gdh* sequences showed that isolate no. 71 from *R. r. rupicapra* clustered within sub-assemblage A/AI, sharing the same sequences as those from a large number of isolates from humans (L40509) [29, 30], several domestic animals, including cattle (EF507642) [31], and also from water (KM190761) [32], whereas isolate no. 10 from *R. p.ornata* clustered within the sub-assemblage AIII, together with a *Giardia* isolate from roe deer in the Netherlands (DQ100288) [29] and red deer in Poland (HM150751) [9] (Table 1; Fig. 2). Bootstrap analysis indicated strong statistical support for these grouping. PCR based on *gdh* locus failed for the other *Giardia* isolates. The nucleotide sequences obtained in this study have been deposited in EMBL/GenBank database under accession number from LN875379 to LN875384 for the *SSU-rDNA* gene and KT270858-KT270859 for the *gdh* gene.

The number of *Giardia* cysts in test-positive samples were predicted to range from 263 to 31,800 per gram of feces (Table 1).

### Ethics

This research did not involve purposeful killing of animals. All fecal samples were gathered from dead free-ranging chamois legally shot by hunters in accordance with the Italian Law (157 of 11/02/1992) which implies that hunters have to carry culled wild ungulates to the control centres where, for each subject, age, sex, the shooting area and morpho-biometric measures are registered. Thus, no animals were killed specifically for this study.

## Discussion

This is the first report of *G. duodenalis* in *Rupicapra* spp. Noticeably, the protist was found both in *R. r. rupicapra* and *R. p. ornata*, two different chamois subspecies living in quite distinct geographical areas, with an overall percentage value of 4.45 % (5.82 and 1.85 %, respectively), and a cyst burden of up to 31,800 cysts/g of feces. Assemblages A (AI and AIII) and E were detected.

Regarding the *G. duodenalis* genetic groups, it is known that assemblage A recognizes four sub-assemblages (AI, AII, AIII and AIV) [3]. Sub-assemblages AI and AII are found in both humans and animals; sub-assemblage AI – the zoonotic subtype – is preferentially found in livestock and pets, but has also been found in wild hoofed animals worldwide [6]; in Europe it has mostly been detected in cervids, i.e. fallow deer (*Dama dama*) in Italy [8], and red deer (*Cervus elaphus*) and roe deer (*Capreolus capreolus*) in Croatia [15]. Sub-assemblage AII is predominantly found in humans, whereas sub-assemblage AIII appears to be specifically associated with wild ungulates [6]; it has been isolated from cervids, i.e. red deer and roe deer in Croatia [15] and Poland [9], but also from cats [5, 33], and in a few cases from cattle [7]. AIV is almost exclusively found in domestic ungulates, and similarly to AIII it is only animal-related; therefore, both sub-assemblages are considered non-zoonotic [6].

Assemblage E is relatively host-specific, or rather ‘group-specific’, since it is limited to ‘hoofed livestock’ i.e. cattle, sheep, goats and pigs [6, 34–36], and for this reason it is known as the ‘livestock genotype’ [6]. However, assemblage E has been also detected in a wild hoofed cervid, i.e. fallow deer [33].

In the present study, the detection of assemblages A and E in chamois living in the Italian Alps and Apennines was not unexpected. It shows that also *R. r. rupicapra* and *R. p. ornata* chamois harbor assemblages/sub-assemblages A/AI/AIII, and confirms that assemblage E is associated to wild hoofed mammals, not only cervids [33] but also wild bovids, such as chamois *Rupicapra r. rupicapra*. In view of this, the term ‘*livestock genotype*’ commonly used to classify genetic group E may be considered outdated and could possibly be replaced with the term ‘*hoofed animal genotype*’.

The presence of both assemblages A/AI and E in Alpine chamois can be related to their sharing of pastures with cattle and/or sheep and/or goats, as well as with cervids. In summer, farmers move their livestock up to high altitude alpine pastures, thus facilitating interaction with wild mountain ungulates [37]. Moreover, in addition to chamois, other species of wild ungulates are present in the alpine areas investigated; therefore, it is not only red deer and roe deer – cervid species found harbouring sub-assemblage AI *Giardia* in Croatia [15]- which may have an epidemiological role for *Giardia* trasmission, but also alpine ibex (*Capra ibex*), a bovid species as yet uninvestigated for the presence of *Giardia*.

Furthermore, unlike other Alpine areas (i.e. the Dolomites), which attract thousands of human visitors involved in trekking and mountaineering, and where “tourist-borne” arrival of *Giardia* may be considered possible [14], the possibility of human-borne contamination by sub-assemblage AI appears unlikely in the *R. r. rupicapra* sampling areas, due to the remoteness of this territory accessible only to a few shepherds in summer and hunters in autumn. This seems to confirm that domestic and wild animals play a greater role in the dissemination of sub-assemblage AI than humans [6].

Conversely, in the Apennine area investigated where one positive chamois was detected (Val di Rose), the *R. p. ornata* population is totally isolated from domestic ruminants and never shares pastures with them [38]; more importantly, red deer and roe deer are present. Both species were reintroduced to ALMNP in 1972–1987 [39]; however, while red deer are present at high densities in the chamois range (with peaks of 0.5–1 deer/ha, in the grasslands of Val di Rose and Mt. Amaro), roe deer density is very limited, at least in summer [21, 38]. Based on this, and coupled with AIII detection in red deer and roe deer in Croatia [15] and in Poland [9], detection of wild ungulate-related sub-assemblage AIII [6] in the Apennine chamois can be fully justified.

Finally, although none of the investigated positive subjects showed signs of diarrhea, since only formed feces were collected, and the *Giardia* cyst burden was up to 31,800 cysts/g of feces, the pathogenic role of *Giardia* in wildlife remains unclear.

## Conclusions

The findings of the present study indicate that *Rupicapra* spp. chamois harbor *G. duodenalis.* This is the first report of assemblage A/AI and assemblage E in *R. r. rupicapra* and AIII in *R. p. ornata*. The epidemiological roles that these wild bovids play in environmental contamination (including watercourses and watersheds) and transmission to other wild and domestic mammals or even humans, of zoonotic (A/AI) and/or non-zoonotic assemblages/sub-assemblages (E, AIII), require further investigation, as does the impact of *Giardia* on the health and sustainability of chamois populations, together with the possible cumulative effects of other pathogens [11].

## Abbreviations

ALMNP: Abruzzo, Lazio and Molise National Park
*q*PCR: Quantitative real time PCR
